# Can Positioning Systems Replace Timing Gates for Measuring Sprint Time in Ice Hockey?

**DOI:** 10.3389/fphys.2018.01882

**Published:** 2019-01-18

**Authors:** Daniel Link, Marcus Weber, Daniel Linke, Martin Lames

**Affiliations:** Department of Exercise Science and Sports Informatics, Technical University Munich, Munich, Germany

**Keywords:** sports analytics, performance analysis, positioning systems, timing gates, validation, on-ice test, smallest worthwhile change

## Abstract

This study explores whether positioning systems are a viable alternative to timing gates when it comes to measuring sprint times in ice hockey. We compared the results of a single-beam timing gate (Brower Timing) with the results of the Iceberg optical positioning system (*Optical*) and two radio-based positioning systems provided by InMotio (*Radio 1*) and Kinexon (*Radio 2*). The testing protocol consisted of two 40 m linear sprints, where we measured sprint times for a 11 m subsection (*Linear Sprint 11*), and a shuttle run (*Shuttle Total*), including five 14 m sprints. The exercises were performed by six top-level U19 field players in regular ice hockey equipment on ice. We quantified the difference between measured sprint times e.g., by Mean Absolute Error (MAE) (s) and Intra Class Correlation (ICC). The usefulness of positioning systems was evaluated by using a Coefficient of Usefulness (CU), which was defined as the quotient of the Smallest Worthwhile Change (SWC) divided by the Typical Error (both in s). Results showed that radio-based systems had a higher accuracy compared to the optical system. This concerned Linear Sprint 11 (MAE_Optical_ = 0.16, MAE_Radio1_ = 0.01, MAE_Radio2_ = 0.01, ICC_Optical_ = 0.38, ICC_Radio1_ = 0.98, ICC_Radio2_ = 0.99) as well as Shuttle Total (MAE_Optical_ = 0.07, MAE_Radio1_ = 0.02, MAE_Radio2_ = 0.02, ICC_Optical_ = 0.99; ICC_Radio1_ = 1.0, ICC_Radio2_ = 1.0). In Shuttle Total, all systems were able to measure a SWC of 0.10 s with a probability of >99% in a single trial (CU_Optical_ = 4.6, CU_Radio1_ = 6.5, CU_Radio2_ = 5.1). In Linear Sprint 11 an SWC of 0.01 s might have been masked or erroneously detected where there were none due to measurement noise (CU_Optical_ = 0.6, CU_Radio1_ = 1.0, CU_Radio2_ = 1.0). Similar results were found for the turning subsection of the shuttle run (CU_Optical_ = 0.6, CU_Radio1_ = 0.5, CU_Radio2_ = 0.5). All systems were able to detect an SWC higher than 0.04 s with a probability of at least 75%. We conclude that the tested positioning systems may in fact offer a workable alternative to timing gates for measuring sprints times in ice hockey over long distances like shuttle runs. Limitations occur when testing changes/differences in performance over very short distances like an 11 m sprint, or when intermediate times are taken immediately after considerable changes of direction or speed.

## Introduction

Performance tests are an important component of training (Wisloff, 2004; Krustrup et al., 2005). They can be used to identify strengths and weaknesses of athletes and to draw conclusions for training and competition (Geithner et al., 2006; Gabbett et al., 2008; Karcher and Buchheit, 2014; Massuca et al., 2015; Suarez-Arrones et al., 2015; La Monica et al., 2016). In ice hockey, sprinting speed is – besides strength and endurance – an important component of physical performance (Bracko, 2001; Roczniok et al., 2016). To measure the individual sprinting performances of athletes, teams mainly use so-called off-ice tests (Leone et al., 2007; Burr et al., 2008; Janot et al., 2015). A well-known example is the *Entry Draft Combine* (Gledhill and Jamnik, 2007; National Hockey League, 2015), which is used by the National Hockey League (NHL) in order to determine the abilities of youth players. The results of the different strength, endurance, coordination and speed tests (e.g., bench press, squat-jumps, Wingate test, balance board, and 40-yd sprint) are used to predict their prospects for a professional career.

Off-ice tests have the advantages of being performable with little organizational effort, that they are easy to standardize, and that there is also a great deal of comparable data available from other team sports (Durandt et al., 2007; Vaz et al., 2014; Slimani and Nikolaidis, 2017). The most critical point is the transferability of results to a specific sport. In terms of sprinting performance, it is questionable whether a 40-yd sprint on turf (Burr et al., 2008) is a good indicator of sprinting performance on ice. The scientific literature does not provide a homogenous picture on this issue. Some studies claim high external validity for off-ice tests (Roczniok et al., 2012; Janot et al., 2015; Henriksson et al., 2016), while other publications doubt the predictive power of such tests for performance on ice (Vescovi et al., 2006; Farlinger et al., 2007; Durocher et al., 2010; Buchheit et al., 2011; Nightingale et al., 2013; Allisse et al., 2017). Certainly, on-ice tests are a more valid way to predict sprinting ability of ice hockey players in the match situation.

As in many other sports, sprint times in ice hockey are usually measured by using timing gates or laser radar (LIDAR) (Türk-Noak, 1994). A comparatively new option is the use of radio-based or optical positioning systems, which have become popular in many sports (Carling et al., 2008; Aughey, 2011; Leser et al., 2011). Nowadays, these systems are often permanently installed in ice hockey venues and are used for collecting physical and tactical performance parameters in training and competition. Whereas the use of timing gates and LIDAR generates additional effort for system set up, this is not the case for positioning systems once they have been installed. Another potential advantage is that tests can be performed on the entire ice surface without a rearrangement of measuring devices. They can also measure speed in non-linear movements and provide high-frequency information about acceleration, which is very important in ice hockey (Buckeridge et al., 2015). GPS-based positioning systems, which are used in many outdoor sports, are not suitable for ice hockey, since the roof at ice-rinks blocks communication with the geostationary satellites.

Against this background, the question arises whether positioning systems offer a viable alternative for timing gates in on-ice speed tests. In particular, when talking about sprints or split times over short distances, there are exacting reliability requirements in order to allay the possibility that small changes or differences in performance are masked by inaccuracies produced by the measurement device (Hopkins, 2004; Haugen and Buchheit, 2016). The scientific literature comprises many studies that evaluate the accuracy of radio-based (Frencken et al., 2010; Ogris et al., 2012; Buchheit et al., 2014; Stevens et al., 2014) and optical positioning systems (Di Salvo et al., 2006; Redwood-Brown et al., 2012; Buchheit et al., 2014; Mara et al., 2017). Parameters under inspection include coordinates on the pitch (Ogris et al., 2012; Siegle et al., 2013; Linke et al., 2018) as well as derived performance indicators, such as running distance, speed, acceleration or time/distances in speed sectors (Di Salvo et al., 2006, 2007; Frencken et al., 2010; Ogris et al., 2012; Buchheit et al., 2014; Castellano et al., 2014; Stevens et al., 2014; Linke et al., 2018).

Some of these studies compare positioning systems with timing gates for average speed (Di Salvo et al., 2006; Frencken et al., 2010; Redwood-Brown et al., 2012; Siegle et al., 2013), but they do not discuss their application as a timing device. To date, there is only one study which deals with the reliability of sprint time measurement using positioning systems (Buchheit et al., 2014). However, this issue is only a marginal consideration in that study. Thus, the question of the extent to which positioning systems are able to substitute timing gates remains unanswered. To our knowledge, all evaluation studies for positioning systems hitherto have used settings with movements on stable ground – movements on ice have never been investigated. This must be considered a deficit since the manufacturers of positioning systems have not optimized the data processing algorithms for gliding movements. Bond et al. (2017b) recently studied the reliability of two timing gates for short sprints in ice hockey, but without considering positioning systems.

The aim of this study was to explore the agreement between sprint times measured by a timing gate with one optical and two radio-based positioning systems in on-ice tests. We questioned here whether these positioning systems are sufficient to determine worthwhile performance changes/differences in typical test settings. As mentioned above, the reliability of sprint time measurement using the first radio system has only been tested in a marginal way (Buchheit et al., 2014). For the second radio system, there is only one study on mechanical sprint properties, e.g., acceleration time, velocity, power output (Hoppe et al., 2018). There is no validation study available for the optical system. The results of this study should therefore contribute to the evaluation of possibilities and limitations of all the above-mentioned systems for their use in ice hockey performance testing.

## Materials and Methods

### Equipment

To answer our research question, we applied an experimental approach. The experiment took place at the Ice Arena in Salzburg, Austria. Positional data was simultaneously collected by three so-called Electronic Performance and Training Systems (EPTS): the Iceberg optical system (Iceberg Hockey Analytics Corp., Toronto, Canada) (*Optical*), which was specially developed for ice hockey, and the two radio-based systems manufactured by InMotio (Inmotiotec GmbH, Regau, Austria) (*Radio 1*) and Kinexon (Kinexon GmbH, Munich, Germany) (*Radio 2*). Firmware versions and application software versions corresponded to the latest releases on the testing date (January 2017). All systems were installed and operated by the manufacturers. Reference sprint times were measured by single-beam timing gates (Brower Timing Systems, IRD-T175), which were located at the face-off spots in the neutral zone. Their precise position was determined using a tachymeter (Leica Geosystems, Viva-TS11). The distance between the timing gates was 11.23 m. The transmitter was located 1.20 m above the ground.

### Exercises

Our testing protocol comprised a linear sprint and a shuttle run. For the linear sprint, the face-off spots in the end zones were used as start and ending points, so that the total sprint distance was about 40 m. The shuttle run included five shuttle sprints and four shuttle turns. For these, the blue lines were used as start and ending points, which resulted in a one-way sprint distance of about 15.5 m. Players had to pass the blue lines with both skates before they change running direction. All players used a standardized standing start position. Between the trials, the players had more than 5 min to recover completely.

### Sample

Six U19 field players from the Ice Hockey Club Red Bull Salzburg, wearing regular ice hockey equipment, executed the test runs. Each player performed two linear sprints and one shuttle run. The protocol was approved by the ethics committee of Technical University Munich. All subjects gave written informed consent in accordance with the Declaration of Helsinki.

**FIGURE 1:**
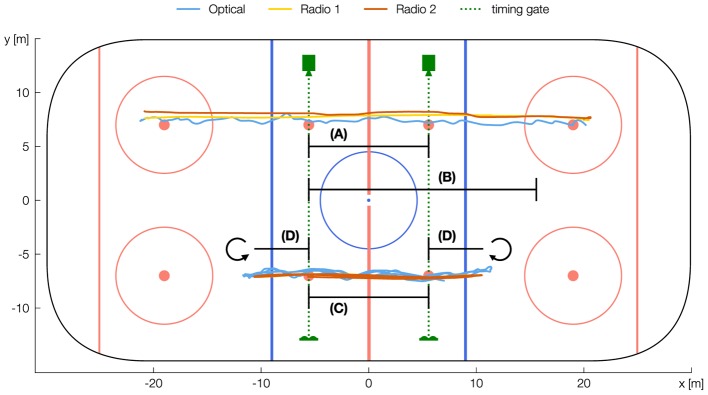
Visualization of test setting and segments Linear Sprint 11 **(A)**, Linear Sprint 21 **(B)**, Shuttle Sprint **(C)**, and Shuttle Turn **(D)**. Shuttle Run Total is given as the concatenation of all Shuttle Sprints and Shuttle Turns. The Figure shows the original trajectory of one athlete measured simultaneously by each EPTS.

### Variables

In each exercise, we collected sprint times in the segments (RUN) *Linear Sprint 11* (between two face-off spots)*, Linear Sprint 21* (between the first face-off spot and the rear face-off circle), *Shuttle Turn* (between face-off spot, turn and same face-off spot), *Shuttle Sprint* (between the two face-off spots) and *Shuttle Total* (entire exercise) (Figure 1). Before calculating sprint times from positional data (SPRINT TIME EPTS), the different sampling rates (Optical: 10 Hz, Radio 1: 100 Hz, Radio 2: 15 Hz) were aligned to 100 Hz using cubic spline interpolation. SPRINT TIME EPTS was determined based on the temporal distance of the data points *Start* and *End*. These are the timestamps of data frames, in which the x-coordinate was closest to the measurement line of the timing gate. Reference time (SPRINT TIME) was measured using the timing gates, except for *Linear Sprint 21*. Here, we calculated SPRINT TIME using the mean of Radio 1 and Radio 2. The measurement error (E) was calculated by the difference of SPRINT TIME and SPRINT TIME EPTS. All sprint times were rounded up to within two decimal places.

To assess the usefulness of the positioning systems, we followed Hopkins (2004) approach. This involves setting the Typical Error (TP), which represents the measurement noise, in relation to the so-called Smallest Worthwhile Change (SWC) and the Change in Performance (CP) measured by the system. The quotient of SWC and TE, which we refer to as Coefficient of Usefulness (CU), was used to derive the probabilities of the CP (which could be affected by measurement noise) being substantial. As per Hopkins, we used the following scale to describe the probability of a substantial change in performance: 25–75% (*possible*), 75–95% (*likely*), 95–99% (*very likely*), and >99% (*almost certain*). Details and examples of this approach are also given by Haugen and Buchheit (2016). The SWC for Shuttle Total (=0.10 s) and for Linear Sprint 21 (=0.02 s) was set to 20% of the between-subject SD of SPRINT TIME (Hopkins et al., 2009); for all other categories of RUN we used an SWC of 0.01 s. For all RUNs except Linear Sprint 21, TE was calculated as the SD of E divided by √2 (Bond et al., 2017a). The TE of Linear Sprint 21 was set to the value of TE for Linear Sprint 11.

**Table 1:** Results of accuracy testing for each RUN and EPTS.

RUN × EPTS	N	ME	MAE	MXE	TE	CV	LOA	ICC	R
**Linear Sprint 11**									
Optical	12	−0.16	0.16	−0.19	0.01	1.2	[−0.2 to −0.12]	0.38	0.97
Radio 1	12	∼0	0.01	−0.03	0.01	0.7	[−0.04 to 0.03]	0.96	0.98
Radio 2	12	−0.01	0.01	−0.02	0.01	0.7	[−0.03 to 0.02]	0.96	0.99
**Shuttle Total**									
Optical	6	−0.07	0.07	−0.12	0.02	0.1	[−0.13 to −0.01]	0.99	0.97
Radio 1	6	0.01	0.02	0.04	0.01	0.1	[−0.03 to 0.05]	1.0	0.97
Radio 2	6	∼0	0.02	0.05	0.02	0.1	[−0.05 to 0.06]	1.0	0.96
**Shuttle Sprint**									
Optical	29	−0.12	0.12	−0.16	0.02	0.8	[−0.16 to −0.07]	0.55	0.99
Radio 1	29	0.05	0.05	0.1	0.02	1.2	[−0.01 to 0.11]	0.86	0.99
Radio 2	29	−0.04	0.04	−0.12	0.02	1.2	[−0.1 to 0.02]	0.89	0.99
**Shuttle Turn**									
Optical	23	0.12	0.12	0.17	0.02	0.7	[0.08 to 0.17]	0.80	1.0
Radio 1	23	−0.06	0.06	−0.11	0.02	0.7	[−0.11 to −0.02]	0.94	1.0
Radio 2	23	0.05	0.05	0.09	0.02	0.8	[−0.01 to 0.1]	0.96	1.0

*Accuracy is presented by mean error (ME), mean absolute error (MAE), maximum error (MXE), typical error (TE) (all in s), coefficient of variation (CV), lines of agreement (LOA), intra class correlation (ICC), and Pearson’s R. ME values between −0.005 s and 0.005 s are reported as ∼0. Analysis excludes one Shuttle Sprint and one Shuttle Turn because of severe tracking errors.*

**FIGURE 2:**
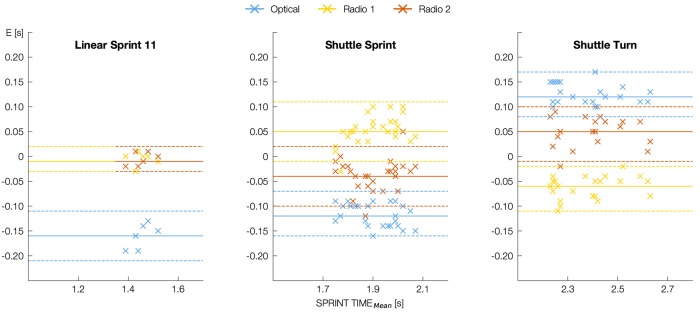
Visualization of accuracy for each trial and each EPTS according to Bland-Altman. The plots show Measurement Error (E) over SPRINT TIME_Mean_ (mean of sprint time measured by EPTS and timing gate) as well as Mean Error (ME, solid colored line) and Lines of Agreement (LOA, dashed colored line) (all in s). Negative values indicate shorter EPTS sprint times compared to the timing gate.

**Table 2:** Indicators for evaluating the usefulness of tested EPTS.

RUN	SPRINT TIME	SWC	CU_Optical_	CU_Radio1_	CU_Radio2_
Linear Sprint 11	1.45 ± 0.05	0.01	0.6^−^	1.0^#^	1.0^#^
Linear Sprint 21	2.63 ± 0.08	0.02	1.1^#^	2.0^+^	2.0^+^
Shuttle Total	19.15 ± 0.48	0.10	4.6^+^	6.5^+^	5.1^+^
Shuttle Sprint	1.90 ± 0.10	0.01	0.6^−^	0.5^−^	0.5^−^
Shuttle Turn	2.41 ± 0.18	0.01	0.6^−^	0.6^−^	0.5^−^

*For each RUN, we report SPRINT TIME. SWC, smallest worthwhile change (both in s); CU, coefficient of usefulness. CUs index indicates the related EPTS. Superscript shows, if usefulness is clearly given (+), at the critical limit (#), or poor (−).*

### Statistical Analysis

**FIGURE 3:**
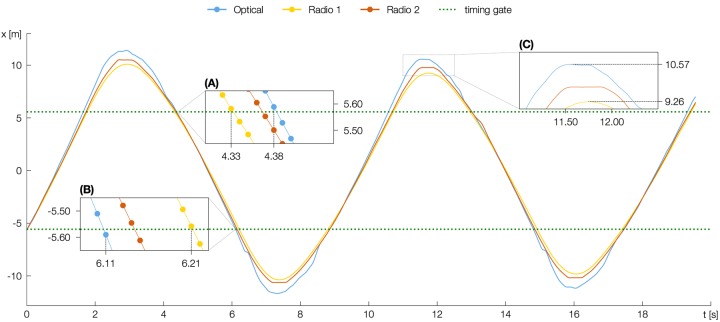
Course of players x-coordinate (in m) over time (in s) during Shuttle Run measured simultaneously by each EPTS. The enlarged segments show when the player passes the measurement line of the timing gates at the end of the Shuttle Turn **(A)**, the end of the Shuttle Sprint **(B)**, and when reaching the turnover moment in the third Shuttle Turn **(C)**. Total SPRINT TIME for this Shuttle Run measured by the timing gates was 19.41 s (SPRINT TIME Optical = 19.30 s, SPRINT TIME Radio 1 = 19.43 s, SPRINT TIME Radio 2 = 19.37 s).

Analysis of accuracy used the Measurement Error of one trial of one player of one RUN and of one EPTS as a statistical unit (*n* = 220). Data is presented in the categories of EPTS × RUN (except for Linear Sprint 21) by reporting Mean Error (ME), Mean Absolute Error (MAE), Maximum Error (MXE), TE (all in s), Coefficient of Variation (CV) (ratio of mean sprint time per run and TE), Intra Class Correlation (ICC) (two way mixed, single measurement model) and Pearson’s R (Table 1). Bland-Altman-Plots (Bland and Altman, 1986) were used for visualization (Figure 2). The effect of EPTS on MAE was tested by a one-factorial ANOVA. For testing differences between two systems, we used post-hoc analysis (Tukey’s test). Before using parametric statistical test procedures, we verified the assumption of normality. The α-level was set to 0.05. All statistical analyses were performed using R (v3.5). Variables for evaluating the usefulness of the systems are presented in the categories of RUN (Table 2). For each EPTS we report SPRINT TIME as the mean ± standard deviation, SWC (both in s) and CU. Graphic analysis of movement trajectory uses the course of x-coordinate over time (Figure 3). The data frame at which the player was closest to the measurement line of the timing gates was used as a trigger point for temporal synchronization of positional data.

## Results

In Linear Sprint 11 (*F* = 416.23, *p* < 0.001), Shuttle Total (*F* = 9.7, *p* < 0.05), Shuttle Sprint (*F* = 74.94, *p* < 0.001) and Shuttle Turn (*F* = 71.07, *p* < 0.001), the MAE was significantly higher for the optical system compared to the radio-based systems (Table 1). In addition, the ICC was smaller for the optical system in all RUNs. It is striking that there was only a moderate correlation between the optical system and the timing gates for Linear Sprint 11 and Shuttle Sprint. Pearson’s *R* shows almost perfect agreements between timing gates and all EPTS. There were no significant differences in the MAE between the two radio-based systems in any setting, and the difference regarding ICC was quite small (maximum 0.03).

In Shuttle Sprint and Shuttle Turn, all three systems displayed slight characteristic variations (Figure 2). In Shuttle Sprint, we measured shorter sprint times with Optical and Radio 2 compared to the timing gates, in Shuttle Turn, sprint times were longer. As an example, Figure 3 shows a part of one athlete’s Shuttle Run with almost identical x-coordinates in the middle of the Shuttle Sprint as measured by the different systems. In Shuttle Turn, there was a difference of up to 1.31 m between the x-coordinates in the turning point (Figure 3C). The optical system detected the passing of the first Shuttle Turns endpoint, which is also the starting point of the second Shuttle Sprint – 0.05 s later as compared to Radio 1 (Figure 3A) – whereas the second endpoint was detected 0.10 s early (Figure 3B). This characteristic variation was evident for all Shuttle Sprints and Shuttle Turns for all athletes.

The CU tended to be smaller in Linear Sprint 11, Shuttle Sprint and Shuttle Turn compared to the longer distances Linear Sprint 21 and Shuttle Total (Table 2). The optical system showed a general tendency to smaller CUs compared to the radio-based systems. Exceptions were found only for Shuttle Sprint and Shuttle Turn, where all systems showed similar values.

## Discussion

### Discussion of Methods

The study aimed to evaluate three positioning systems with respect to their validity and usefulness for measuring sprint times in ice hockey. We therefore used the sprint times measured by timing gates as the ground truth. These results are limited by the fact that timing gates have an inherent error. According to the International Association of Athletics Federations (IAAF, 2017), the end of a sprint is defined as the moment at which one part of the torso passes the vertical plane above the finish line. For flying starts, this definition can also be applied to the start line. One of the drawbacks of timing gates is that they can be triggered by swinging arms and legs. The size of their measurement error depends on the way the body passes the measurement line and is influenced by starting technique, running speed and posture, height of the photocells and the type of the timing gate (Cronin and Templeton, 2008; Haugen et al., 2012; Haugen et al., 2014; Altmann et al., 2017; Bond et al., 2017a). For the single-beam timing gate used in this study, Bond et al. (2017a) reported a MAE = 0.02 s and a TE = 0.01 s for 20–30 ft split times compared with high-speed cameras, which is comparable to the situation in Linear Sprint 11. The same study showed measurement errors for 10–20 ft split times, which correspond to Shuttle Sprint and Shuttle Turn, of MAE = 0.03 s and TE = 0.03 s. For ice hockey, we expect equal or less measurement errors, since the sliding movement in ice hockey has observationally a more frontal than sagittal component (e.g., swinging arms and legs in the frontal plane) compared to running. However, this potential error would not affect the direction of our findings.

Another limitation concerns the way sprint times were measured via ETPS. Positioning systems usually work with different internal sampling rates, which are limited, among other things, by the number of registered sensors or the power of the video processing units. In our study, we used the raw data provided by the manufacturers, which were recorded with different sampling rates. In order to derive a sprint time with two digits and to enable comparison of the difference, we up-sampled the data to 100 Hz. Our procedure only provides an estimation of the moment in which the player passes the measurement line – it is not a direct measurement. It is possible that other internal sampling rates or advanced interpolation methods could influence the results.

To assess the usefulness of positioning systems for measuring sprint times, we refer to the SWC-model (Hopkins, 2004), which uses the SWC defined for each specific test. Estimation of the SWC can be based on statistical spread and/or observations of direct benefits for performance (Hopkins et al., 2009). Following Hopkins’ recommendation, we used 0.2 × SD of the performance variable for Shuttle Run and Linear Sprint 21 (SWC_ShuttleTotal_ = 0.10 s, SWC_LinearSprint21_ = 0.02 s). This value is influenced by the group homogeneity, but is widely accepted as a standard in the scientific community. The purpose of Linear Sprint 21 in this study was to introduce an additional performance variable, which allows evaluation of the usefulness of a setting, where the SWC is between Linear Sprint 11 and Shuttle Total. Therefore, it was not necessary to use timing gates for this segment.

Whereas Shuttle Run and Linear Sprint 21 are primarily used in test settings, short sprints play an important role in ice hockey, particularly when players are dueling for the puck. For this reason, we argue for performance benefits over statistical considerations when choosing an SWC of 0.01 s for Linear Sprint 11, Shuttle Sprint and Shuttle Turn. Following Haugen et al. (2014) for soccer, we assume that the minimal distance needed to win the puck is around its diameter (7.62 cm). This distance corresponds to a time difference of 0.01 s at the end of a 10 m sprint. Duthie et al. (2006) also use this value for 10 m sprints in rugby.

The SWC-model also requires determining the TE, which represents the measurement noise of a performance test. Research in this field usually uses the mean value of the within-subject variation of the performance variable for this (Hopkins, 2004; Darrall-Jones et al., 2016; Bond et al., 2017b). In this study, we applied an alternative option and took the random part the measurement error of positioning systems as TE (Bond et al., 2017a). Consequently, our assessment of usefulness asks, whether the technical noise is low enough to detect the SWC and not, whether the noise of the performance variable (including also biological fluctuation) is low enough to detect the SWC. Although this is a limitation, because reference is made only the technical properties of the test, this approach is adequate for technology-centered evaluations.

The results of the study might be limited by the quite small number of participants. This affects the reliability of the SWC estimation, especially for Shuttle Total. Nevertheless, a moderate change of SWC would not affect our findings significantly, since CUs for Shuttle Total are quite high. In addition, TE could differ on the individual level due to variability of movement techniques and anthropometric factors. However, we find no traces of such effects in our data.

### Discussion of Results

The results show that agreement between sprint times measured by the timing gate and the positioning systems (i) depends on the positioning system and (ii) is influenced by the exercise in question. The radio-based systems we tested showed better agreement compared to the optical system, which is in line with previous studies (Siegle et al., 2013; Linke et al., 2018). One explanation for this could be that radio-based systems use differences between the reception times of a sensor signal at different receiving stations (Stelzer et al., 2004), whereas optical systems rely on color, contour and movement information in video footage (Xu et al., 2004; Link, 2014). In video, position is determined by a blob of pixels, which allows only a rough estimation of the center of gravity. In any case, this finding is just a snapshot of current state of technology available and things could change with the next generation of optical systems (e.g., by using higher optical resolutions).

We can explain the different trajectories in Shuttle Run (Figure 3), which have a direct impact on the measurement error, by different post-processing procedures. Positioning systems usually do not provide the system-internal raw data as output but as a processed value resulting from the application of various filter and smoothing operations. One of these is the Kalman filter (Kalman, 1960), which estimates the position based on current measured coordinates, and direction and speed of preceding moments. In this way, noise and outlier values can be eliminated. Kalman filters also have the disadvantage that rapid changes in speed and direction are reproduced with time delay and less acuity. As a consequence, output data show a system-specific characteristic according to the filter parameters used, which also leads to differences in derived performance indicators, line running distances, or time spent in speed intervals (Frencken et al., 2010; Ogris et al., 2012; Siegle et al., 2013; Linke et al., 2018).

For positioning systems, various studies have shown that reliability declines with less running distance, higher speed, and an increasing number of direction changes (Coutts and Duffield, 2010; Jennings et al., 2010; Portas et al., 2010; Rawstorn et al., 2014; Linke et al., 2018). These findings are also supported by our data. We can explain the a) smaller ICC of the optical system when it comes to higher speed (Linear Sprint 11, Shuttle Sprint), b) the higher MAE in Linear Sprint 11 and c) the stronger amplitude of the trajectory during a Shuttle Turn by a higher measurement error and larger time windows for filtering. The radio-based systems also had problems reproducing changes of direction and speed during the Shuttle Run. Here, the signal showed a “delay” (Figure 3), which was caused by the Kalman filter and led to higher deviations from the timing gates compared to Linear Sprint 11. It might be worth adopting the approach taken by Buchheit et al. (2014) and find correction factors and filter parameters for each system, which reduce measurement errors for one specific test setting. For Linear Sprint 11, the correlation between the radio-based systems and the timing gates are high and the measurement errors are very small – without a clear tendency to overestimation or underestimation. These results seem to be compatible with the ones reported by Buchheit et al. (2014) for Radio 1, although their procedures remain somewhat vague.

According to the SWC-model, a system is ‘useful’ for a test if the CU is greater than 1.0. If this is granted, we can use the CU values showed in Table 2 to assign all categories of EPTS x RUN to one of three groups: a group U^+^, where the system is clearly useful, a group U^#^ where usefulness is at the critical limit, and a group U^−^ in which usefulness is poor in terms of detecting the SWC. In Shuttle Total, the probability of a measured change of CP = 0.10 s being true is >99% and can be classified as *almost certain.* A change of CP = 0.02 s measured by the radio-based systems in Linear Sprint 21 is substantial with a probability of >75% (*likely*). The same probability can be assumed for the optical system when CP = 0.04 s. All these combinations belong to group U^+^. In contrast to this, the chance of all systems reliably detecting an SWC of 0.01 s in Shuttle Sprint or Shuttle Turn and for Optical in Linear Sprint 11 is marginal, since such small changes of performance could be masked or falsely recorded on account of measurement errors. It is important to understand that this applies only if an SWC of 0.01 s is used – an SWC of 0.04 s, for example, could be detected with a probability of more than 75%.

All these interpretations underlie the condition of accepting timing gates as a ground truth. In the light of their limitations (Duthie et al., 2006; Haugen et al., 2014; Bond et al., 2017a), other studies conclude that timing gates are only of marginal use for measuring SWC for sprints over ∼10 m (Duthie et al., 2006; Darrall-Jones et al., 2016). Because of the high agreement between timing gates and the radio-based systems in Linear Sprint 11, we adhere to this argumentation and recommend interpreting time changes of ≤0.02 s measured by positioning systems – as well as measured from timing gates – with a certain degree of caution. Further investigations might also consider the noise of the performance variable in order to get a different perspective on the usefulness of positioning systems.

Nevertheless, our results provide a good argument for accepting these systems as an alternative to timing gates for measuring sprint time in linear sprints. We can also speculate as to which system class is better suited here. An argument for radio-based systems would be that they obviate the swinging legs or arms problem, as the sensor is located between the shoulders. On the other hand, their underlying measurement principle is much more complex than detecting a light beam interruption. Previous studies on Radio 1 report an average spatial error of 0.23 m (Ogris et al., 2012; Linke et al., 2018), which would lead to a measurement error of around 0.03 s in detecting the crossing of the measurement line in Linear Sprint 11. Since the error compared with the timing gates is much smaller in this study, one could argue that positional errors are compensated for as long as they are systematic and the movement between start and finish line is linear. In the end, both system classes have different advantages and disadvantages for measuring sprint times.

## Conclusion

The positioning systems we tested may be a viable alternative for using timing gates for measuring sprint time in ice hockey. Compared to timing gates, they have several advantages since they are also able to measure accelerations and provide performance indicators in non-linear movement tests. In addition, the application of positioning systems requires far fewer resources once they have been installed. The validity and usefulness of the positioning systems tested depends on the test exercise. All systems were able to detect an SWC higher 0.04 s with a probability of at least 75%. They are therefore useful for measuring sprint performance in exercises over longer distances like a Shuttle Run. Limitations occur when testing changes/differences in performance over very short distances (e.g., an 11 m sprint), or when intermediate times are taken right after considerable changes of direction or speed. In linear sprints, the radio-based systems are more useful than the optical system, but these systems also demonstrate – thus as with timing gates – only a marginal chance of detecting a worthwhile change of around 0.01 s.

## Compliance With Ethical Standards

Board approval for their research and appropriate consent has been obtained pursuant to law.

## Author Contributions

DLk contributed to the conception and design of the study and wrote the manuscript. MW performed the statistical analysis and data visualization. DLe worked on data visualization. All authors contributed to manuscript revision, read, and approved it for publication.

## Conflict of Interest Statement

The authors declare that the research was conducted in the absence of any commercial or financial relationships that could be construed as a potential conflict of interest.
